# Privacy-Preserving Task-Matching and Multiple-Submissions Detection in Crowdsourcing

**DOI:** 10.3390/s21093036

**Published:** 2021-04-26

**Authors:** Jie Xu, Zhaowen Lin, Jun Wu

**Affiliations:** 1 The School of Cyberspace Security, Beijing University of Posts and Telecommunications, Beijing 100876, China; 2 The National Engineering Laboratory for Mobile Network Security, Beijing 100876, China; linzw@bupt.edu.cn (Z.L.); wujun@bupt.edu.cn (J.W.); 3 The School of Computer Science, Beijing University of Posts and Telecommunications, Beijing 100876, China

**Keywords:** task-matching, anonymous multi-submission detection, inner-product encryption, zero-knowledge proof

## Abstract

Crowdsourcing enables requesters to publish tasks to a platform and workers are rewarded for performing tasks of interest. It provides an efficient and low-cost way to aggregate data and solve problems that are difficult for computers but simple for humans. However, the privacy risks and challenges are still widespread. In the real world, the task content may be sensitive and only workers who meet specific requirements or possess certain skills are allowed to acquire and perform it. When these distributed workers submit their task answers, their identity or attribute privacy may also be exposed. If workers are allowed to submit anonymously, they may have the chance to repeat their answers so as to get more rewards. To address these issues, we develop a privacy-preserving task-matching and multiple-submissions detection scheme based on inner-product cryptography and proof of knowledge (PoK) protocol in crowdsourcing. In such a construction, multi-authority inner-product encryption is introduced to protect task confidentiality and achieve fine-grained task-matching based on the attributes of workers. The PoK protocol helps to restrict multiple submissions. For one task, a suitable worker could only submit once without revealing his/her identity. Moreover, different tasks for one worker are unlinkable. Furthermore, the implementation analysis shows that the scheme is effective and feasible.

## 1. Introduction

With the development of network technologies and the popularity of smartphones, crowdsourcing has become a popular distributed paradigm for problem-solving, which is applied to address problems that are too complex for computer programs or of high cost for an organization. An early typical example of crowdsourcing is captcha. ReCAPTCHA [1], a project initiated by Carnegie Mellon University, uses the wisdom of the masses to help the digitization of ancient books in the form of crowdsourcing. This project scans the text, which cannot be recognized by the optical character recognition technology accurately, and displays it in the captcha question, so that a human can recognize it when answering the captcha question.

In the era of big data, the amount of data is increasing, and the forms of data are more diversified, which leads to increasing demand for crowdsourcing and the increasing forms of tasks. Crowdsourcing platforms such as Amazon Mechanical Turk (AMT) [2], crowdflower and upwork of Amazon came into being. On these platforms, tens of millions of workers from more than 100 countries are involved in solving problems. It has inspired the collective imagination of researchers in numerous fields such as human–computer interaction, machine learning, artificial intelligence, information retrieval, database community, etc.

The openness and sharing of crowdsourcing make it more vulnerable to various attacks since it allows attackers to join crowdsourcing systems freely as requesters or workers. When task requesters have tasks to crowdsource, they need to set some parameters, including task pricing, answer time, task worker quality, etc. After that, they can publish tasks on the crowdsourcing platform, and then the tasks will be assigned to workers. When a task is answered by a worker, the requester can choose to accept or reject the answer. If the requester accepts the answer, he/she will pay the worker accordingly. In this process, combined with the task constraints, task content and worker authentication information, the attacker may infer the important private information of the participants, including identity, age, occupation, residence, and so on. If such kind of information cannot be properly kept, it will reduce the enthusiasm of users to participate in the task and further affect the completion of the task.

In the process of task release and matching, since different workers have their own specialties, unsuitable or malicious workers may randomly answer questions to get the reward, or deliberately submit wrong answers to distort the true value. To ensure the quality of answers, the requester should set up task constraints for different tasks so as to match appropriate workers. There are many ways of keyword matching. The flexibility of accurate matching is poor. The matching method that supports multiple policy expressions is more in line with diverse requirements, e.g., ((major = (art ∨ artificial intelligence)) ∧ (age ≥ 30)), etc. Under the premise of privacy protection, how to achieve flexible task-matching has become a thorny issue.

In most previous mutual privacy-preserving task allocation research, the homomorphism [3] is adopted to realize multiple types of ciphertext policy matching without revealing task constraints and workers’ private attributes, which cause the downgrade of efficiency. Moreover, content confidentiality is closely related to the privacy of participants. For the privacy of the task content, the proxy re-encryption algorithm or other technologies is needed. Then the computation and communication cost is further increased. However, based on inner-product encryption the relevant work [4] considered flexible matching of encrypted keywords and fine-grained access control of task content simultaneously. With the expansion of the network scale, it is difficult for a single authentication center to manage workers’ keys efficiently. The multi-authority model [5] could better adapt to a large-scale distributed network. However, at this time, there are not only collusion problems of workers but also collusion or damage problems of some attribute authorities in the system.

After the task is assigned, the worker will perform the task and submit task data. At this phase, we should first ensure that it is the right workers who meet the requirements submit the answers. However, similarly, the workers may not wish to be tracked by the server. Since the platform is not completely credible, it may expose the worker’s privacy because of interest-driven. Due to the flexible matching requirements set by the task requester, an attribute-based signature could be used. It allows signers to sign a message under policies that satisfying their attributes. In a crowdsourcing system, the worker obtains the attribute-based private key from the authority. When his/her attributes satisfy the constraint policy set by the requester, the signature can be verified to be valid. With anonymous attribute-based signature authentication, it is possible to prevent inappropriate workers from submitting, while avoiding the leakage of worker’s privacy. However, dishonest qualified workers may submit multiple answers to a task for more rewards. Moreover, if a greedy participant submits similar or identical results with different pseudonyms many times, it will also reduce the diversity and credibility of the data, and further produce bias to the results that should have been perceived by numerous participants. Actually, a privacy-preserving submission detection scheme is needed, which ensures that only qualified workers can participate in answering and cannot submit repeatedly, and the worker’s identity and history of participating in the task will not be disclosed.

In this paper, we first analyze the potential security threats to the privacy and quality assurance issues of crowdsourcing during the task allocation and task submission phase, and then propose a security and privacy protection model of the system. After that, a scheme based on multi-authority inner-product encryption (MIPE) and zero-knowledge proof protocol, called zk-MIPE, is designed. With MIPE, the scheme can realize secure sharing of task content and the flexible assignment of tasks based on encrypted task constraints and workers’ attributes. With the repeated submission detection algorithm constructed by zero-knowledge proof protocol, it ensures that the requester and platform can only verify that a worker who has submitted an answer about a task meets the corresponding task constraints but cannot judge his/her specific identity or attribute information. Also, if the worker performs multiple tasks, no one can link them. At the same time, when workers submit repeatedly for the same task, they can be identified by association. Under the premise of protecting the participants’ privacy, the scheme selects suitable workers to submit an answer honestly with more professional skills, thus further improve the quality of aggregated task data. In summary, the technical innovation of the proposed system is: we designed a novel MIPE scheme and a one-time anonymous inner-product authentication protocol based on zero-knowledge proof, and proved the confidentiality, one-time authentication, anonymity and unlinkability of the solution. In terms of application, we achieved the innovative features in function and security for crowdsourcing privacy protection: (1). it supports flexible task-matching based on inner-product with mutual privacy; (2). it supports anonymously inner-product-based authentication and duplicate submission detection without revealing identity and attributes privacy.

## 2. Related Work

*Crowdsourcing Privacy*

Presently, for a variety of data processing and analysis tasks, only relying on machine algorithms cannot achieve desired results. Fortunately, crowdsourcing provides an efficient and low-cost paradigm to solve this problem with the advantage of distributed mode. However, security and privacy issues are still thorny. In past research on privacy-preserving, some researchers analyzed the privacy threats of the whole crowdsourcing process to propose an overall security framework [6]. Meanwhile, blockchain is applied to deal with potential security issues (e.g., single point of failure, sensitive leakage) without a trusted third party, such as SecBCS [7], MCS-chain [8], CrowdBC [9]. Also, novel fog-based computing framework is proposed [10] for low latency vehicular crowdsensing networks.

Still, there are researchers in-depth discussing crowdsourcing security threats at each phase, and designing differentiated privacy protection schemes for specific security objectives using diversified technologies. Among them, location privacy is the first concern of researchers. The methods used to solve location privacy include k-anonymity [11], differential privacy [12,13], game theory [14], commitment [15], machine-learning-based obfuscation [16,17], encryption [18,19], etc. However, most of them focus on protecting the workers’ privacy. To provide mutual privacy for both requesters and workers, Liu [3] proposed a privacy-preserving protocol based on homomorphic encryption with a dual-server setting. After that, Shu [20] constructs a task-matching scheme over the encrypted location with a single server by applying searchable encryption. Actually, in the scenes they mainly concern, the privacy requirements of task content are not high, which are usually public to all participants. However, the need for content privacy protection still exists. For some sensitive task content involving address, occupation and purpose, it can help attackers to further infer participants’ privacy by combining other information. In the privacy-aware task assignment schemes proposed by Liu et al. [21] and Yuan et al. [22], attribute-based encryption is applied to protect content privacy and realize fine-grained access control. Extending to more complex multi-keyword crowdsourcing allocation scenario, our prior work [4] introduced inner-product encryption (IPE) to support flexible matching policies without disclosing task privacy and worker privacy. However, as the worker scale increases, centralized single authority mode has obvious disadvantages in efficiency and security.

Moreover, most of these schemes mainly discussed privacy protection in the task allocation phase. While in the data submission phase, the platform should verify the identity or attribute information of the participants to evaluate whether the appropriate workers have performed the task. At this time, if we do not provide effective privacy protection, the secure closed-loop still cannot be constructed. Based on signature and other technologies, Ni [23] and Shu [24] presented Sybil detection schemes respectively. Nevertheless, they are concerned about the deduplication of encrypted data content rather than the identity privacy of workers. Though Lu [25] proposed a blockchain-based private and anonymous repetition detection scheme for task submission, the introduction of zk-SNARK increases the computational overhead of the scheme. Compared with the previous scheme, we focus on the privacy protection of task releasing and task submission. In the task releasing stage, the scheme requires privacy of task content and constraint conditions, and should realize flexible ciphertext task-matching. In the task submission phase, workers could submit perceptual data anonymously and cannot submit it repeatedly.

*Inner-Product Cryptosystems*

In 1984, Shamir [26] proposed the concept of ID-based public key cryptography and constructed the first ID-based digital signature scheme based on the large integer decomposition problem. However, it was not until 2001 that Boneh and Franklin [27] presented the first secure and practical ID-based encryption scheme based on elliptic curve bilinear pairings. After that, Sahai and Waters [28] designed a fuzzy identity-based encryption scheme based on key sharing theory in 2005, and further proposed the concept of attribute-based encryption (ABE). Since then, research on ABE has covered privacy protection, richer access policy types, efficiency, security assumptions, attribute revocation, and other directions [29,30,31]. To implement policy hiding, Boneh and Waters [32] introduced a hidden vector encryption scheme supporting conjunctive, subset and range queries in 2007. Then Katz [33] raised the concept of IPE for the first time and proved its security under the standard model in 2008. The scheme allows conjunctive disjunction, polynomial and inner-product queries. However, the length of ciphertext increases linearly with the increase of vector dimension. Afterwards, Attrapadung and Libert [34] developed a scheme to reduce the length of ciphertext to a constant. Furthermore, Okamoto [35] realized a scheme with constant key length. On the other hand, to reduce the management cost of a single authentication server, Chase [5] presented an encryption scheme that enables the implementation of the AND access policy in a multi-authority environment. On this basis, to reduce the complexity of user decryption, Li [36] constructed a multi-authority outsourcing attribute encryption system based on linear secret-sharing schemes (LSSS). However, the IPE scheme in multi-authority environment still needs to be proposed. For anonymous authentication, Yuen [37] adopted *k* times attribute signature (*k*-ABS) to restrict access times. The data is still stored remotely in plaintext. Ning presented an outsourced σ-time attribute-based encryption (σ-ABE) scheme [38], in which users apply attributes as identity without using real names. Although the server cannot know a user’s identity, it can associate a user’s previous and subsequent access through the proxy key. Moreover, due to the lack of association between the attribute-related private key and the validation tags for times, there is a risk that the attacker will steal the other’s validation tag, and then send his own attribute-related private key to access the data illegally. Inner-product cryptosystems enables the realization of flexible and diverse policies. Compared with cryptosystems supporting LSSS policy, it allows policy hiding. However, presently, neither the IPE encryption for multi-authority nor the k-time inner-product-based authentication scheme has been proposed. Therefore, in this article, we intend to solve this problem and apply the design scheme to crowdsourcing privacy protection.

## 3. Preliminary

### 3.1. System Assumption

**Figure 1:**
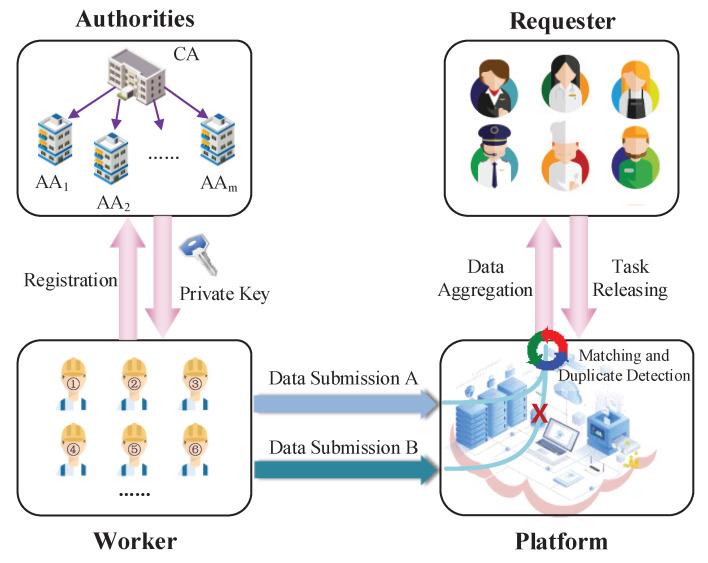
Framework of the zk-MIPE system.

As shown in Figure 1, the proposed crowdsourcing system contains the following entities: central authority CA, multiple attribute authorities AA, the crowdsourcing server CS, requesters and workers. As a trusted third party, CA initializes the system, generates global parameters and supervises each AA. Suppose there are *m* attribute authorities, denoted as $AA_{1}$,...,$AA_{m}$. They are responsible for managing disjoint attribute sets. The requester is an enterprise or individual who publishes the task on the system platform. The worker is a user who performs tasks and submits perception data. CS verifies whether workers meet the requirements and submit repeatedly. Let the sets of vectors $\vec{w}=\left(w_{1},...,w_{m}\right)\in Z_{q}^{mn}$ and $\vec{z}=\left(z_{1},...,z_{m}\right)\in Z_{q}^{mn}$ be the task constraint and the worker’s attribute-based vector. Only if $<z_{j},w_{j}>=0$ holds for all j∈[1,m], the worker could decrypt the corresponding task ciphertext.

For system security, we need at least one attribute authority is honest and secure in such a system. The requester is also considered to be honest. CS is considered to be honest-but-curious, i.e., it will honestly execute the protocol and screen out suitable workers, but it will also be curious about more information, such as task content and participant identity. The worker is considered to be honest but greedy, i.e., he will execute the protocol honestly but may submit data multiple times to get more rewards.

The specific security objectives of the zk-MIPE scheme are as follows.

(1) *Content and constraints privacy.* Task content and constraints should be released in the form of ciphertext. Only suitable workers could learn the corresponding task plaintext.

(2) *One-time attribute-based authentication.* If the worker’s attributes meet the task constraints, he/she can provide a valid proof to the CS. If not, he/she cannot forge a valid proof.

(3) *Identity and attribute privacy.* Although the CS enables the filtering out of suitable workers and the restriction of multiple submission, it cannot know the worker’s identity and attributes, or even associate the previous and subsequent tasks that a worker participates in.

### 3.2. Inner-Product Access Structure

The inner product is a generalization of the concept of point multiplication. In a vector space, it is a method of multiplying vectors, and the product is a scalar. For a real vector space, let $x_{1},x_{2},x_{3}$ be vectors and *r* be a scalar, then the inner product <·,·> satisfies the following properties.

(1) $<x_{1}+x_{2},x_{3}>=<x_{1},x_{3}>+<x_{2},x_{3}>$;

(2) $<rx_{1},x_{2}>=r<x_{1},x_{2}>=<x_{1},rx_{2}>$;

(3) $<x_{1},x_{2}>=<x_{2},x_{1}>$;

(4) $<x_{1},x_{1}>\geq 0$, and only when $x_{1}=0$ the equal sign holds.

### 3.3. Bilinear Group

**Definition** **1.** *Bilinear Map [27]: A group generator G takes a security parameter λ as input. It outputs a group $\vec{G}=\left(G_{1},G_{T},e,q\right)$ of prime order q, where $G_{1}$ is an additional group and $G_{T}$ is a multiplication group. Let g be a generator of $G_{1}$. The bilinear map e has the following properties.*

*(1) Bilinearity: For random $a,b\in Z_{q}$ and $x,y\in G_{1}$, we have $e\left(x^{a},y^{b}\right)=e{\left(x,y\right)}^{ab}$;*

*(2) Nondegeneracy: e(g,g)≠1;*

*(3) Computability: For random $g,h\in G_{1}$, there exists an efficient algorithm to compute e(g,h).*

**Definition** **2.** *Computational Diffie-Hellman (CDH) Problem: A challenger runs G(λ) to generate $\vec{G}=\left(G_{1},G_{T},e,q\right)$. Then it chooses a random generator g and random $a,b\in Z_{q}$. Given a tuple $\left(g,g^{a},g^{b}\right)$ as input, we say that the CDH assumption holds if there is no polynomial-time algorithm can compute the element $g^{ab}$.*

**Definition** **3.** *Decisional Diffie-Hellman (DDH) Problem: A challenger runs G(λ) to generate $\vec{G}=\left(G_{1},G_{T},e,q\right)$. Then it chooses a random generator g and random $a,b\in Z_{q}$. Given a tuple $\left(g,g^{a},g^{b}\right)$ as input, we say that the DDH assumption holds if there is no polynomial-time algorithm can distinguish $g^{ab}$ from a random value with nonnegligible advantage in $G_{1}$.*

**Definition** **4.** *q-Decisional Diffie-Hellman Inversion (DDHI) Problem: A challenger runs G(λ) to generate $\vec{G}=\left(G_{1},G_{T},e,q\right)$. Then it chooses a random generator g and a random $x\in Z_{q}$. Given a tuple $\left(g,g^{x},g^{x^{2}},...,g^{x^{q}}\right)$ as input, we say that the q-DDHI assumption holds if there is no polynomial-time algorithm can distinguish $g^{1/x}$ from a random value with nonnegligible advantage in $G_{1}$.*

### 3.4. Zero-Knowledge Proof Protocol

The zero-knowledge proof (ZKP) protocols have been applied to numerous fields, including both traditional secure multiparty computation and emerging privacy protection projects in distributed ledger and blockchain, such as Zcash [39], hawk [40], and so on.

A ZKP system is a protocol between a computationally bounded prover and a verifier. Let *R* be an NP relation. Set R(x)={w:(x,w)∈R} and the language L={x:∃w,st(x,w)∈R}. During the protocol, the verifier is convinced by the prover that *x* belongs to *L*, i.e., there exists a witness *w* such that (x,w)∈R for x. However, in proof of knowledge (PoK), the prover cannot only prove the exists of some witness but also be convinced that he/she indeed know a specific witness *w*.

The main properties of ZKP for a relation *R* are as follows.

*Completeness*: Given a witness ω that satisfies (x,ω)∈R, the prover could convince the verifier of his knowledge. i.e.,

Verify(Prove(x,*w*)) = accept.

*Soundness*: Given a witness ω that does not satisfy (x,ω)∈R, for any polynomial-time prover, the probability that the verification can be accepted is negligible. i.e.,

Pr[Verify(Prove(x,*w*)) = accept∧(x,*w*) ∉ *R*] ≤ *neg* (λ).

*Zero knowledge*: The interaction between a prover and a verifier is called a view. The zero-knowledge property could be captured by the existence of a simulator *E* that could access to the verifier’s input but not the prover’s: with the assumption x∈L, if the simulated view, i.e., the transcript, is indistinguishable from the original view between the honest prover and the verifier, whether honest or cheating. We say the ZKP scheme has the property of zero knowledge. Moreover, in PoK, there exists a knowledge extractor, which has rewindable access to the prover, and could extract the witness with nonnegligible probability.

## 4. Model of zk-MIPE

**Definition** **5.** *A privacy-preserving task-matching and multi-submission detection scheme zk-MIPE is defined by a tuple of the following algorithms:*

- **CA Setup**(λ,m). The algorithm is executed by the central authority CA. It takes a security parameter λ and several attribute authorities *m* as inputs. It then publishes a system public key PK and keeps a system master key SK secretly.
- **AA Setup**(λ,n). Run by the attribute authorities $AA_{j}$, the algorithm takes a security parameter λ and several intra-domain attributes *n* as inputs. It then outputs a public key $PK_{j}$ and an attribute-related secret key $SK_{j}$ for each $AA_{j}$.
- **Task Releasing**$\left(M,PK,{\left\{PK_{j}\right\}}_{j\in [1,m]},\vec{w}\right)$. Executed by the requester, the algorithm takes the public key, a message and a constraint as inputs. Then it outputs an inner-product ciphertext *C*.
- **Registration**$\left(SK,u,{\left\{SK_{j}\right\}}_{j\in [1,m]},\vec{z}\right)$. According to the identity *u* and attributes $\vec{z}$, the secret key $K_{u}$ for the registrant is generated by CA and $AA_{j}$.
- **Decryption**$\left(C,K_{u}\right)$. Executed by the worker *u*, the algorithm takes the ciphertext *C* and the private key $K_{u}$ as inputs. It then outputs the message *M*.
- **Matching and Multi-Submission Verification**$\left(C,{\left\{PK_{j}\right\}}_{j\in [1,m]},K_{u}\right)$. Executed by CS and workers, this algorithm takes as inputs the public parameter $PK_{j}$, the private key $sk_{j}$ and the ciphertext *C*. It then runs a zero-knowledge proof to verify the compliance of attributes and submission times between CS and the worker. It then outputs accept or reject.

## 5. zk-MIPE Scheme

**Figure 2:**
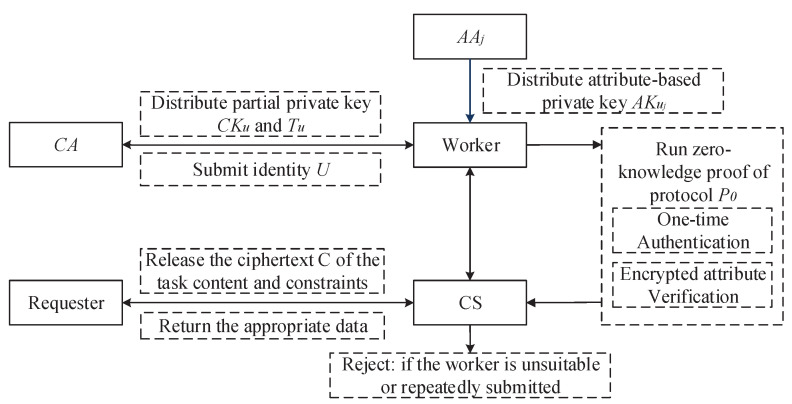
Overview of the zk-MIPE Scheme.

Based on the difficult problems of bilinear pairings and a specific zero-knowledge proof protocol, we propose a zk-MIPE algorithm to deliver task-matching and multiple data submissions detection services in crowdsourcing. The scheme is roughly described in Figure 2.

For instance, suppose the task content is to collect some physical indicators, and the task constraint is: male, 48 years old, and suffering from hypertension or arthritis. Let $Z_{1}$, $Z_{2}$, $Z_{3}$ be three attributes, which represent gender, age and disease. Let $z_{1}$, $z_{2}$, $z_{3}$ be the specific attribute values for workers. We quantify gender and disease in task constraints, e.g., for attribute $Z_{1}$, set male = 1 and female = 2, and for attribute $Z_{3}$, set hypertension = 1, arthritis = 2, gastritis = 3. Then the restriction is $\left\{Z_{1}=1\wedge Z_{2}=45\wedge \left(Z_{3}=1\vee Z_{3}=2\right)\right\}$ which could be further denoted as $r_{1}\left(Z_{1}-1\right)+r_{2}\left(Z_{2}-45\right)+r_{3}\left(Z_{3}-1\right)\left(Z_{3}-2\right)=0$ for $r_{1},r_{2},r_{3}\in F_{q}$. The worker’s attribute vector $\vec{z}$ is defined as $\left(1,z_{1},z_{2},z_{3},z_{3}^{2}\right)$. To make the equation $r_{1}\left(z_{1}-1\right)+r_{2}\left(z_{2}-45\right)+r_{3}\left(z_{3}-1\right)\left(z_{3}-2\right)=0$ hold if and only if the inner product $<\vec{w},\vec{z}>=0$ is zero, the vector $\vec{w}$ is defined as $\left(-45r_{1}-r_{2}+2r_{3},r_{1},r_{2},-3r_{3},r_{3}\right)$.

Given a task ciphertext encrypted with restriction $\vec{w}$, if a worker’s attribute is: male, 45 years old, with hypertension, he will be able to decrypt the task ciphertext and be eligible to participate in the task. In the task submission stage, he could generate a proof in the form of zero-knowledge and sends it to the CS together with his collected data. In the process of verification, the CS can verify whether the worker meets the constraints and whether the submission is repeated, but cannot get the explicit attribute information of the worker. Each worker could select a random number ϕ as his identity-based private key. For each task, he sends the calculated $S=e{\left(g,g\right)}^{\frac{1}{\phi +H(C_{task})}}$, where *H* is a one-way hash function, and the proof of the attribute private key to the CS. Through a zero-knowledge proof protocol he will prove to the CS that it is the first time to submit, and he is a suitable worker without disclosing $\phi ,\vec{z}$, and the private key of $\vec{z}$. The value of *S* is unique for one task. If the CS detects the same *S*, it means duplicate submission. Moreover, if a worker chooses another random number $\phi^{\prime }$ as his identity-based private key, since the attribute private key, generated by the authorities, is bound with the information of ϕ, he will not be able to pass the verification of matching attributes and constraints.

Furthermore, a crowdsourcing task usually involves multiple workers. IPE just solves the problem of one to many. A ciphertext can be decrypted by many users, which is suitable for multi-user scenarios. Once the crowdsourcing requester encrypts a task, it can be decrypted by any worker who meets the requirements. For the crowdsourcing server with mighty computing power, it is also feasible to handle the task requests issued by multiple requesters in parallel. The introduction of multiple authorities further increases the scalability of the scheme.

Specifically, the scheme is as follows.

- **CA Setup**(λ,m). Executed by CA, the algorithm takes a security parameter λ as input and runs G(λ) to output a symmetric group $\vec{G}=\left(G_{1},G_{T}\right)$ of prime order *q*. It picks a random generator $g\in G_{1}$, a random $t\in Z_{q}$ and a one-way hash function $H_{1}:{\left\{0,1\right\}}^{*}\to G_{1}$. Then it sets the public key as $PK=\{g,Y=g^{t},H_{1}\}$ and the system master key as SK={t}.
- **AA Setup**(λ,n). The attribute authority ${AA}_{j}$ randomly picks $\alpha_{j},\gamma_{j1},...,\gamma_{jn}\in Z_{q}$ and computes $h_{ji}=g_{1}^{\gamma_{ji}}$ as the public key for each attribute ${Att}_{ji}$ belonging to ${AA}_{j}$. Then $AA_{j}$ publishes $PK_{j}=\left\{e{\left(g_{1},g_{1}\right)}^{\alpha_{j}},h_{j1},...h_{jn}\right\}$ and sets ${SK}_{j}=\left\{\alpha_{j},\gamma_{j1},...\gamma_{jn}\right\}$ as its secret key.
- **Task Releasing**$\left(M\in G_{T},PK,{\left\{PK_{j}\right\}}_{j\in [1,m]},\vec{w}=\left(w_{1},...,w_{m}\right)\in Z_{q}^{mn}\right)$. The algorithm, executed by the requester, takes the public key PK, ${PK}_{j}$ (for j∈[1,m]), description of constraints $\vec{w}=\left(w_{1},...,w_{m}\right)\in Z_{q}^{nm}$ in which $w_{j}=\left(w_{ji}\right)\in Z_{q}^{n}$ and the message $M\in G_{T}$ as input. It randomly chooses $s_{1},s_{2},\sigma_{1},\sigma_{2}\in Z_{p}$ and computes
  $$C_{0}=M\cdot e{\left(g,g\right)}^{\sum_{j=1}^{m}s_{1}\alpha_{j}},C_{1}=g^{s_{1}},C_{2}=Y^{s_{1}},C_{1}^{{}^{\prime }}=g^{s_{2}},C_{2}^{{}^{\prime }}=Y^{s_{2}},$$
  $${\left\{C_{ji}=h_{ji}^{s_{1}}\cdot g^{\sigma_{1}w_{ji}},C_{ji}^{{}^{\prime }}=h_{ji}^{s_{2}}\cdot g^{\sigma_{2}w_{ji}}\right\}}_{i\in [1,n],j\in [1,m]},C_{T}=e{\left(g,g\right)}^{\sum_{j=1}^{m}s_{2}\alpha_{j}}.$$
  Then it outputs the task ciphertext as
  $$C=(C_{0},C_{1},C_{2},C_{1}^{{}^{\prime }},C_{2}^{{}^{\prime }},C_{11},C_{11}^{{}^{\prime }},...,C_{mn},C_{mn}^{{}^{\prime }},C_{T}).$$
- **Registration**$\left(SK,u,{\left\{SK_{j},z_{j}=\left(z_{j1},...,z_{j_{n}}\right)\in Z_{q}^{n}\right\}}_{j\in [1,m]}\right)$. Users can either register as requesters or workers. Both CA and $AA_{j}$ are responsible for generating private keys for registered users by calling the following algorithms.

(1) If a user registers as a worker, he/she first selected a random $\phi \in Z_{q}$, computes $U=g^{\phi }$ as the public key, and sends *U* to CA. Then CA randomly picks $\beta_{u_{1}},...,\beta_{u_{m}}\in Z_{q}$, sets $\beta_{u}=\sum_{j=1}^{m}\beta_{u_{j}}$ and distributes $\beta_{u_{j}}$ to $AA_{j}$ secretly. In particular, $\beta_{u}$ corresponds uniquely with the worker *u*. Then CA computes
  $$CK_{u}=g^{\frac{\beta_{u}}{t}},T_{u}={\left(Ug\right)}^{\frac{t}{\beta_{u}+t}}.$$
  After that, CA sends $\left(CK_{u},T_{u}\right)$ to the worker. For each registered requester, CA sends the system public key to the requester.
(2) After receiving $\beta_{u_{j}}$ from CA, $AA_{j}$ chooses a random $\tau_{u_{j}}\in Z_{q}$ and computes $Q_{u_{j}}=g^{\tau_{u_{j}}}$ for the worker *u*. Then it creates the secret key about the attribute-based vector $z_{j}$ as
  $${AK}_{u_{j}}=g^{\alpha_{j}-\beta_{u_{j}}}\cdot Q_{u_{j}}^{\sum_{i=1}^{n}\gamma_{ji}z_{ji}}.$$
  The algorithm outputs the worker secret key as $K_{u}=\left(CK_{u},T_{u},{\left\{AK_{u_{j}},Q_{u_{j}}\right\}}_{j\in [1,m]}\right)$.

- **Decryption**$\left(C,K_{u}\right)$. The algorithm, executed by the worker, takes the ciphertext *C* and the secret key $K_{u}$ as input. It first computes
  $$E_{1}=e\left(CK_{u},C_{2}\right),E_{2}=\prod_{j=1}^{m}\frac{e(AK_{u_{j}},C_{1})}{\prod_{i=1}^{n}e(Q_{u_{j}}^{z_{ji}},C_{ji})}$$.
  Then it could recover message *M* by computing $M=\frac{C_{0}}{E_{1}\cdot E_{2}}$.
  When $<\vec{w},\vec{z}>=0$, the computation is correct since
  $$E_{1}=e\left(CK_{u},C_{2}\right)=e\left(g^{\frac{\beta_{u}}{t}},g^{ts_{1}}\right)=e{\left(g,g\right)}^{\beta_{u}s_{1}},$$
  $$\begin{array}{c} \begin{array}{cc} \; & E_{2}=\prod_{j=1}^{m}\frac{e(AK_{u_{j}},C_{1})}{\prod_{i=1}^{n}e(Q_{u_{j}}^{z_{ji}},C_{ji})}=\prod_{j=1}^{m}\frac{e(g^{\alpha_{j}-\beta_{u_{j}}}\cdot Q_{u_{j}}^{\sum_{i=1}^{n}\gamma_{ji}z_{ji}},g^{s_{1}})}{\prod_{i=1}^{n}e(Q_{u_{j}}^{z_{ji}},h_{ji}^{s_{1}}g^{\sigma_{1}w_{ji}})}\\ \; & =\prod_{j=1}^{m}\frac{e\left(g^{\alpha_{j}-\beta_{u_{j}}},g^{s_{1}}\right)\cdot e\left(Q_{u_{j}}^{\sum_{i=1}^{n}\gamma_{ji}z_{ji}},g^{s_{1}}\right)}{\prod_{i=1}^{n}e\left(Q_{u_{j}}^{z_{ji}},h_{ji}^{s_{1}}\right)e\left(Q_{u_{j}}^{z_{ji}},g^{\sigma_{1}w_{ji}}\right)}\\ \; & =e{\left(g,g\right)}^{\sum_{j=1}^{m}\left(\alpha_{j}-\beta_{u_{j}}\right)s_{1}}\cdot \prod_{j=1}^{m}\frac{e(Q_{u_{j}}^{\sum_{i=1}^{n}\gamma_{ji}z_{ji}},g^{s_{1}})}{e{\left(Q_{u_{j}},g\right)}^{s_{1}\sum_{i=1}^{n}\gamma_{ji}z_{ji}}e{\left(Q_{u_{j}},g\right)}^{\sigma_{1}<z_{j},w_{j}>}}\\ \; & =e{\left(g,g\right)}^{\sum_{j=1}^{m}\alpha_{j}s_{1}}\cdot e{\left(g,g\right)}^{{-\beta }_{u}s_{1}}. \end{array} \end{array}$$
  Thus, $\frac{C_{0}}{E_{1}\cdot E_{2}}=\frac{C_{0}}{e{\left(g,g\right)}^{\sum_{j=1}^{m}s_{1}\alpha_{j}}}=M$.
- **Matching and Multi-Submission Verification**$\left(C,{\left\{PK_{j}\right\}}_{j\in [1,m]},K_{u}\right)$. The algorithm tasks the system public, the worker secret key $K_{u}$ and the task ciphertext *C* as inputs. In the interaction protocol between the worker and the platform, if $<w_{j},z_{j}>=0$ for j=[1,m], the worker *u* first computes $S=e{\left(g,g\right)}^{\frac{1}{\phi +H(C_{0})}}$ and sends *S* to CS. Then CS checks whether *S* has been used once. If used, CS rejects the request. If not, CS will allow *u* to run the following zero-knowledge proof of knowledge protocol $P_{0}$ with it to prove the knowledge of $\left(\phi ,K_{u}\right)$:
  $$\begin{array}{cc} \; & P_{0}\{\left(\phi ,K_{u}=\left(CK_{u},T_{u},{\left\{AK_{u_{j}},Q_{u_{j}}\right\}}_{j\in [1,m]}\right)\right):\\ \; & S=e{\left(g,g\right)}^{\frac{1}{\phi +H(C_{0})}}\wedge \\ \; & e\left(T_{u},g\cdot CK_{u}\right)=e\left(Ug,g\right)\wedge \\ \; & e\left(CK_{u},C_{2}^{{}^{\prime }}\right)\cdot \prod_{j=1}^{m}\frac{e(AK_{u_{j}},C_{1}^{{}^{\prime }})}{\prod_{i=1}^{n}e(Q_{u_{j}}^{z_{ji}},C_{ji}^{{}^{\prime }})}=C_{T}\}. \end{array}$$
  To implement the protocol $P_{0}$, *u* will calculate some auxiliary inputs and use some tricks to convert the protocol equivalently. Specifically, *u* interacts with CS as follows.
  (1) CS randomly picks two generators $\hat{g},\hat{h}\in G$ and sends them to *u*, where the discrete logarithm of $\hat{h}$ with respect to $\hat{g}$ is unknown to *u*. Then *u* picks random $\kappa ,\delta ,\mu ,\nu_{1},...,\nu_{m},\varsigma_{11},...,\varsigma_{mn}\in Z_{q}$ and computes
  $$\begin{array}{cc} \; & \pi_{T}=T_{u}{\hat{h}}^{\kappa },\chi_{T}={\hat{h}}^{\delta }{\hat{g}}^{\kappa },\pi_{C}=CK_{u}{\hat{h}}^{\mu },{\left\{\pi_{A_{j}}=AK_{u_{j}}{\hat{h}}^{\nu_{j}}\right\}}_{j\in [1,m]},\\ \; & {\left\{\pi_{Q_{ji}}=Q_{u_{j}}^{z_{ji}}{\hat{h}}^{\varsigma_{ji}}\right\}}_{j\in [1,m],i\in [1,n]},\rho_{1}=\kappa \mu ,\rho_{2}=\delta \mu . \end{array}$$
  After that, *u* returns auxiliary values $\left(\pi_{T},\chi_{T},\pi_{C},{\left\{\pi_{A_{j}},\pi_{Q_{ji}}\right\}}_{j\in [1,m],i\in [1,n]}\right)$ to CS. In this case, the protocol can be expressed as the following zero-knowledge proof of knowledge protocol $P_{1}$ to prove the knowledge of $\left(\phi ,\kappa ,\delta ,\mu ,\nu_{1},...,\nu_{m},\varsigma_{11},...,\varsigma_{mn},\rho_{1},\rho_{2}\right)$:
  $$\begin{array}{cc} \; & P_{1}\{\left(\phi ,\kappa ,\delta ,\mu ,\nu_{1},...,\nu_{m},\varsigma_{11},...,\varsigma_{mn},\rho_{1},\rho_{2}\right):\\ \; & \chi_{T}^{-\mu }\cdot {\hat{h}}^{\rho_{2}}\cdot {\hat{g}}^{\rho_{1}}=1_{G}\wedge \\ \; & S=e{\left(g,g\right)}^{\frac{1}{{}^{\phi +H(C_{0})}}}\wedge \\ \; & e\left(\pi_{T},g\pi_{C}\right)=e\left(\pi_{T},{\hat{h}}^{\mu }\right)\cdot e\left({\hat{h}}^{\kappa },g\pi_{C}\right)\cdot e{\left(\hat{h},\hat{h}\right)}^{-\rho_{1}}\cdot e\left(Ug,g\right)\wedge \\ \; & e\left(\pi_{C},C_{2}^{{}^{\prime }}\right)\cdot \prod_{j=1}^{m}\frac{e(\pi_{A_{j}},C_{1}^{{}^{\prime }})}{\prod_{i=1}^{n}e(\pi_{Q_{ji}},C_{ji}^{{}^{\prime }})}=C_{T}\cdot e{\left(\hat{h},C_{2}^{{}^{\prime }}\right)}^{\mu }\cdot \prod_{j=1}^{m}\frac{e{\left(\hat{h},C_{1}^{{}^{\prime }}\right)}^{\nu_{j}}}{\prod_{i=1}^{n}e{\left(\hat{h},C_{ji}^{{}^{\prime }}\right)}^{\varsigma_{ji}}}\}. \end{array}$$
  Assume that the auxiliary value calculated by *u* has been sent to CS. Next, we will describe the implementation details of the honest-verifier zero-knowledge protocol $P_{1}$ below.
  (1) **Commitment**. *u* picks random $\xi_{\phi },\xi_{\kappa },\xi_{\delta },\xi_{\mu },\xi_{\nu_{1}},...,\xi_{\nu_{m}},\xi_{\varsigma_{11}},...,\xi_{\varsigma_{mn}},\xi_{\rho_{1}},\xi_{\rho_{2}}\in Z_{q}$ and computes
  $$\begin{array}{cc} \; & L_{1}={\hat{h}}^{\xi_{\delta }}{\hat{g}}^{\xi_{\kappa }},L_{2}=\chi_{T}^{-\xi_{\mu }}\cdot {\hat{h}}^{\xi_{\rho_{{}_{2}}}}{\hat{g}}^{\xi_{\rho_{{}_{1}}}},L_{3}=S^{\xi_{\phi }},\\ \; & L_{4}=e{\left(\pi_{T},\hat{h}\right)}^{\xi_{\mu }}\cdot e{\left(\hat{h},g\pi_{C}\right)}^{\xi_{\kappa }}\cdot e{\left(\hat{h},\hat{h}\right)}^{-\xi_{\rho_{{}_{1}}}}\cdot e\left(g,g\right)\cdot e{\left(g,g\right)}^{\xi_{\phi }},\\ \; & L_{5}=C_{T}\cdot e{\left(\hat{h},C_{2}^{{}^{\prime }}\right)}^{\xi_{\mu }}\cdot \prod_{j=1}^{m}\frac{e{\left(\hat{h},C_{1}^{{}^{\prime }}\right)}^{\xi_{\nu_{{}_{j}}}}}{\prod_{i=1}^{n}e{\left(\hat{h},C_{ji}^{{}^{\prime }}\right)}^{\xi_{\varsigma_{{}_{ji}}}}}. \end{array}$$
  Then the worker sends these auxiliary values $L_{1},...,L_{5}$ to CS.
  (2) **Challenge**. CS picks a random $\varepsilon \in Z_{q}$ and sends ε to the worker.
  (3) **Response**. the worker computes the following auxiliary value at first.
  $$\begin{array}{cc} \; & z_{\phi }=\xi_{\phi }-\varepsilon \phi ,z_{\kappa }=\xi_{\kappa }-\varepsilon \kappa ,\\ \; & z_{\delta }=\xi_{\delta }-\varepsilon \delta ,z_{\mu }=\xi_{\mu }-\varepsilon \mu ,\\ \; & z_{v_{1}}=\xi_{\nu_{1}}-\varepsilon \nu_{1},...,z_{v_{m}}=\xi_{\nu_{m}}-\varepsilon \nu_{m},\\ \; & z_{\varsigma_{11}}=\xi_{\varsigma_{11}}-\varepsilon \varsigma_{11},...,z_{\varsigma_{mn}}=\xi_{\varsigma_{mn}}-\varepsilon \varsigma_{mn},\\ \; & z_{\rho_{1}}=\xi_{\rho_{1}}-\varepsilon \rho_{1},z_{\rho_{2}}=\xi_{\rho_{2}}-\varepsilon \rho_{2}. \end{array}$$
  Then *u* sends the sets of $\left(z_{\phi },z_{\kappa },z_{\delta },z_{\mu },z_{v_{1}},...,z_{v_{m}},z_{\varsigma_{11}},...,z_{\varsigma_{mn}},z_{\rho_{1}},z_{\rho_{2}}\right)$ to CS.
  (4) **Verification**. CS checks whether the following equation holds:
  $$\begin{array}{cc} \; & L_{1}={\hat{h}}^{z_{\delta }}\chi_{T}^{\varepsilon }{\hat{g}}^{z_{\kappa }},L_{2}=\chi_{T}^{-z_{\mu }}{\hat{h}}^{z_{\rho_{{}_{2}}}}{\hat{g}}^{z_{\rho_{{}_{1}}}},\\ \; & L_{3}=S^{z_{\phi }-\varepsilon H\left(C_{0}\right)}\cdot e{\left(g,g\right)}^{\varepsilon },\\ \; & L_{4}=e{\left(\pi_{T},\hat{h}\right)}^{\xi_{\mu }}\cdot e{\left(\hat{h},g\pi_{C}\right)}^{\xi_{\kappa }}\cdot e{\left(\hat{h},\hat{h}\right)}^{-\xi_{\rho_{{}_{1}}}}\cdot e\left(g,g\right)\cdot e{\left(g,g\right)}^{\xi_{\phi }},\\ \; & L_{5}={C_{T}}^{1-\varepsilon }\cdot {\left[e\left(\pi_{C},C_{2}^{{}^{\prime }}\right)\cdot \prod_{j=1}^{m}\frac{e(\hat{h},C_{1}^{{}^{\prime }})}{\prod_{i=1}^{n}e(\pi_{Q_{ji}},C_{ji}^{{}^{\prime }})}\right]}^{\varepsilon }\cdot e{\left(\hat{h},C_{2}^{{}^{\prime }}\right)}^{z_{\mu }}\cdot \prod_{j=1}^{m}\frac{e{\left(\hat{h},C_{1}^{{}^{\prime }}\right)}^{z_{\nu_{{}_{j}}}}}{\prod_{i=1}^{n}e{\left(\hat{h},C_{ji}^{{}^{\prime }}\right)}^{z_{\varsigma_{{}_{ji}}}}}. \end{array}$$
  Through the above interactive process, CS verifies whether the workers meet the constraints and submit repeatedly. If the verification is valid, CS returns the task answer submitted by the worker to the requester. As follows, we discuss the soundness of the protocol.
  *Soundness of $P_{0}$*: $P_{1}$ is a 3-move protocol, where the prover sends the commitment, the verifier chooses a random challenge, and the prover response to the challenge based on elliptic curve discrete logs. It is straightforward to show that $P_{1}$ is of soundness, i.e., there exists an extractor $E_{1}$, which is given rewindable black-box access to the prover, could output some witness $\left(\phi ,\kappa ,\delta ,\mu ,\nu_{1},...,\nu_{m},\varsigma_{11},...,\varsigma_{mn},\rho_{1},\rho_{2}\right)$ or a halting symbol ⊥ to indicate "failure". By running $P_{1}$ and calling $E_{1}$, we can construct an extractor $E_{0}$. When $E_{1}$ outputs ⊥, $E_{0}$ outputs ⊥ and stops. If $E_{1}$ outputs the witness $\left(\phi ,\kappa ,\delta ,\mu ,\nu_{1},...,\nu_{m},\varsigma_{11},...,\varsigma_{mn},\rho_{1},\rho_{2}\right)$, the extractor could further output some valid witness $\left(\phi ,\pi_{T},\pi_{C},\pi_{A_{1}},...,\pi_{A_{m}},\pi_{Q_{11}},...,\pi_{Q_{mn}}\right)$ with the same probability. Based on the outputs of $E_{1}$, $E_{0}$ computes
  $$\begin{array}{cc} \; & {\hat{\pi }}_{T}=\pi_{T}{\hat{h}}^{-\kappa },{\hat{\pi }}_{C}=\pi_{C}{\hat{h}}^{-\mu },{\left\{{\hat{\pi }}_{A_{j}}=\pi_{A_{j}}{\hat{h}}^{-\nu_{j}}\right\}}_{j\in [1,m]},\\ \; & {\left\{{\hat{\pi }}_{Q_{ji}}=\pi_{Q_{ji}}{\hat{h}}^{-\varsigma_{ji}}\right\}}_{j\in [1,m],i\in [1,n]}. \end{array}$$
  We show how these values satisfy the equation relation of $P_{0}$ as follows.
  Due to soundness of $P_{1}$,
  $$e\left(\pi_{T},g\cdot \pi_{C}\right)=e\left(\pi_{T},{\hat{h}}^{\mu }\right)\cdot e\left({\hat{h}}^{\kappa },g\cdot \pi_{C}\right)\cdot e{\left(\hat{h},\hat{h}\right)}^{-\rho_{1}}\cdot e\left(Ug,g\right).$$
  Rearranging the terms, where $\rho_{1}=\kappa \mu$:
  $$e\left(\pi_{T},g\pi_{C}\right)\cdot e\left({\hat{h}}^{-\kappa },g\pi_{C}\right)\cdot e\left(\pi_{T},{\hat{h}}^{-\mu }\right)\cdot e{\left(\hat{h},\hat{h}\right)}^{\rho_{1}}=e\left(Ug,g\right).$$
  That is
  $$\begin{array}{cc} \; & e\left(\pi_{T},g\pi_{C}\right)\cdot e\left({\hat{h}}^{-\kappa },g\pi_{C}\right)\cdot e\left(\pi_{T},{\hat{h}}^{-\mu }\right)\cdot e{\left(\hat{h},\hat{h}\right)}^{\rho_{1}}\\ \; & =e\left(\pi_{T}{\hat{h}}^{-\kappa },g\pi_{C}\right)\cdot e\left(\pi_{T}{\hat{h}}^{-\kappa },{\hat{h}}^{-\mu }\right)\cdot e\left({\hat{h}}^{\kappa },{\hat{h}}^{-\mu }\right)\cdot e{\left(\hat{h},\hat{h}\right)}^{\kappa \mu }\\ \; & =e\left({\hat{\pi }}_{T},g{\hat{\pi }}_{C}\right)=e\left(Ug,g\right). \end{array}$$
  Due to soundness of $P_{1}$,
  $$e\left(\pi_{C},C_{2}^{{}^{\prime }}\right)\cdot \prod_{j=1}^{m}\frac{e(\pi_{A_{j}},C_{1}^{{}^{\prime }})}{\prod_{i=1}^{n}e(\pi_{Q_{ji}},C_{ji}^{{}^{\prime }})}=C_{T}\cdot e{\left(\hat{h},C_{2}^{{}^{\prime }}\right)}^{\mu }\cdot \prod_{j=1}^{m}\frac{e{\left(\hat{h},C_{1}^{{}^{\prime }}\right)}^{\nu_{j}}}{\prod_{i=1}^{n}e{\left(\hat{h},C_{ji}^{{}^{\prime }}\right)}^{\varsigma_{ji}}}.$$
  Rearranging the terms:
  $$e\left(\pi_{C},C_{2}^{{}^{\prime }}\right)\cdot \prod_{j=1}^{m}\frac{e(\pi_{A_{j}},C_{1}^{{}^{\prime }})}{\prod_{i=1}^{n}e(\pi_{Q_{ji}},C_{ji}^{{}^{\prime }})}\cdot e{\left(\hat{h},C_{2}^{{}^{\prime }}\right)}^{-\mu }\cdot \prod_{j=1}^{m}\frac{e{\left(\hat{h},C_{1}^{{}^{\prime }}\right)}^{-\nu_{j}}}{\prod_{i=1}^{n}e{\left(\hat{h},C_{ji}^{{}^{\prime }}\right)}^{-\varsigma_{ji}}}=C_{T}.$$
  That is
  $$\begin{array}{cc} \; & e\left(\pi_{C},C_{2}^{{}^{\prime }}\right)\cdot \prod_{j=1}^{m}\frac{e(\pi_{A_{j}},C_{1}^{{}^{\prime }})}{\prod_{i=1}^{n}e(\pi_{Q_{ji}},C_{ji}^{{}^{\prime }})}\cdot e{\left(\hat{h},C_{2}^{{}^{\prime }}\right)}^{-\mu }\cdot \prod_{j=1}^{m}\frac{e{\left(\hat{h},C_{1}^{{}^{\prime }}\right)}^{-\nu_{j}}}{\prod_{i=1}^{n}e{\left(\hat{h},C_{ji}^{{}^{\prime }}\right)}^{-\varsigma_{ji}}}\\ \; & =e\left(\pi_{C}{\hat{h}}^{-\mu },C_{2}^{{}^{\prime }}\right)\cdot \prod_{j=1}^{m}\frac{e(\pi_{A_{j}}{\hat{h}}^{-\nu_{j}},C_{1}^{{}^{\prime }})}{\prod_{i=1}^{n}e(\pi_{Q_{ji}}{\hat{h}}^{-\varsigma_{ji}},C_{ji}^{{}^{\prime }})}\\ \; & =e\left({\hat{\pi }}_{C},C_{2}^{{}^{\prime }}\right)\cdot \prod_{j=1}^{m}\frac{e({\hat{\pi }}_{A_{j}},C_{1}^{{}^{\prime }})}{\prod_{i=1}^{n}e({\hat{\pi }}_{Q_{ji}},C_{ji}^{{}^{\prime }})}=C_{T}. \end{array}$$
  Then $E_{0}$ could output $\left(\phi ,{\hat{\pi }}_{T},{\hat{\pi }}_{C},{\hat{\pi }}_{A_{1}},...,{\hat{\pi }}_{A_{m}},{\hat{\pi }}_{Q_{11}},...,{\hat{\pi }}_{Q_{mn}}\right)$ as the witnesses satisfying $P_{0}$. Therefore $P_{0}$ is of soundness.

## 6. Security Proof

In this section, we analyze the security of our scheme and show that it has the properties of task confidentiality, one-time authentication and anonymity.

Assume there exists a PPT adversary A that wins the following games in our scheme, we can construct a PPT simulator B that solves the CDH problem, DDH problem or the *q*-DDHI problem with nonnegligible advantage.

**Theorem** **1.** *Assume the DDH assumption holds, then the proposed zk-MIPE scheme is IND-CPA secure.*

**Proof.**  Against an adversary who wants to learn task content, the security algorithms are designed as follows. □

**Algorithm I**

- **Init.** The challenger sets $\vec{G}=\left(G_{1},G_{T}\right)$ and randomly chooses $\left(g,g^{a},g^{b},g^{c}\right)\in G_{1}$. It flips a coin $\bar{b}$ outside of $B_{1}$’s view and sets *T* as follows:
  If $\bar{b}=0$, it computes $T=e{\left(g,g\right)}^{abc}$; otherwise, it chooses a random $T\in G_{2}$. Then it sends $\left(g,g^{a},g^{b},g^{c},T\right)$ to $B_{1}$. After that, $A_{1}$ submits the challenge access structure ${\vec{w}}^{*}=\left(w_{1}^{*},...,w_{m}^{*}\right)$ to $B_{1}$.
- **CA Setup.** Given the secure parameter λ, $B_{1}$ randomly chooses $t\in Z_{q}$ and sets $Y=g^{t}$. Then it gives the public key $PK=\{Y,H_{1}\}$ to $A_{1}$.
- **AA Setup.**$B_{1}$ randomly chooses ${\left\{x_{j},\eta_{ji}\right\}}_{j\in [1,m],i\in [1,n]}$ at first. Here, we suppose $AA_{\hat{j}}$ is one of the honest attribute authority.

(1) For $j\neq \hat{j}$, $B_{1}$ sets $\alpha_{j}=x_{j}$, $\gamma_{ji}=\eta_{ji}$ and lets $SK_{j}={\left\{\alpha_{j},\gamma_{ji}\right\}}_{i\in [1,n]}$ for $AA_{j}$. Then it computes $h_{ji}=g^{\eta_{ji}}$ and sends the public key $PK_{j}=\left\{e{\left(g,g\right)}^{\alpha_{j}},h_{j1},...,h_{jn}\right\}$ to $A_{1}$.
(2) For $j=\hat{j}$, $B_{1}$ sets $\alpha_{\hat{j}}=ab+x_{\hat{j}}$, $\gamma_{ji}=-w_{\hat{j}i}^{*}b+\eta_{\hat{j}i}$ and lets $SK_{\hat{j}}={\left\{\alpha_{\hat{j}},\gamma_{\hat{j}i}\right\}}_{i\in [1,n]}$ for $AA_{\hat{j}}$. Then it computes $h_{\hat{j}i}=g^{-w_{\hat{j}i}^{*}b+\eta_{\hat{j}i}}$ and sends $PK_{\hat{j}}=\left\{e{\left(g,g\right)}^{\alpha_{\hat{j}}},h_{\hat{j}1},...,h_{\hat{j}n}\right\}$ to $A_{1}$.

- **Registration Queries I.**$A_{1}$ repeatedly makes registration queries with respect to attribute key value $\vec{z}$ such that $<w_{\hat{j}}^{*},z_{\hat{j}}>\neq 0$. Notice that for any other honest $AA_{j}$, $B_{1}$ will also respond the corresponding secret key even if $<w_{j}^{*},z_{j}>=0$.
  $A_{1}$ chooses a user *u* and sets *U* as his/her public key. It sends *U* to $B_{1}$. Then $B_{1}$ chooses random $\beta_{u_{1}},...,\beta_{u_{m}}\in Z_{q}$, sets $\beta_{u}=\sum_{j=1}^{m}\beta_{u_{j}}$ and computes $CK_{u}=g^{\frac{\beta_{u}}{t}}$, $T_{u}={\left(Ug\right)}^{\frac{t}{\beta_{u}+t}}$. After that $B_{1}$ computes the attribute related secret key as follows.

(1) For $j\neq \hat{j}$, $B_{1}$ chooses a random $\tau_{u_{j}}\in Z_{q}$ and computes
  $$Q_{u_{j}}=g^{\tau_{u_{j}}},AK_{u_{j}}=g^{\alpha_{j}-\beta_{u_{j}}}\cdot Q_{}^{\sum_{i=1}^{n}\gamma_{ji}z_{ji}}.$$
(2) For $j=\hat{j}$, $B_{1}$ randomly chooses a $\tau_{u_{\hat{j}}}\in Z_{q}$ and computes
$Q_{u_{\hat{j}}}=g^{\frac{a}{<w_{\hat{j}}^{*},z_{\hat{j}}>}+\tau_{u_{\hat{j}}}},AK_{u_{\hat{j}}}=g^{\alpha_{\hat{j}}-\beta_{u_{\hat{j}}}}\cdot Q_{u_{\hat{j}}}^{\sum_{i=1}^{n}\gamma_{\hat{j}i}z_{\hat{j}i}}=g^{x_{\hat{j}}-\beta_{u_{\hat{j}}}+\frac{a<\eta_{\hat{j}},z_{\hat{j}}>}{<w_{\hat{j}}^{*},z_{\hat{j}}>}-b\tau_{u_{\hat{j}}}<w_{\hat{j}}^{*},z_{\hat{j}}>+\tau_{u_{\hat{j}}}<\eta_{\hat{j}},z_{\hat{j}}>}.$

- **Challenge.**$A_{1}$ submits two challenge messages $M_{0},M_{1}\in G_{T}$ to $B_{1}$. $B_{1}$ flips a coin b∈{0,1} and computes the ciphertext as follows.
  $B_{1}$ chooses a random $\left(\varphi ,s_{2}\right)\in Z_{q}$, sets $s_{1}=c+\varphi$ and computes
  $C_{0}^{*}=M_{b}\cdot e{\left(g,g\right)}^{\sum_{j=1}^{m}s_{1}\alpha_{j}}=M_{b}\cdot T\cdot e{\left(g,g\right)}^{\sum_{j=1}^{m}\left(c+\varphi \right)x_{j}+ab\varphi }$, $C_{1}^{*}=g^{s_{1}}=g^{c+\varphi }$, $C_{2}^{*}=Y^{s_{1}}=Y^{c+\varphi }$, $C_{1}^{{}^{\prime }*}=g^{s_{2}}$, $C_{2}^{{}^{\prime }*}=Y^{s_{2}}$, $C_{ji}^{{}^{\prime }*}=h_{ji}^{s_{2}}g^{\sigma_{2}w_{ji}^{*}}=g^{\eta_{ji}s_{2}+\sigma_{2}w_{ji}^{*}}$, $C_{T}^{*}=e{\left(g,g\right)}^{\sum_{j=1}^{m}s_{2}\alpha_{j}}$.
  Then $B_{1}$ computes $C_{ji}^{*}$ as follows.

(1) For $j\neq \hat{j}$, $B_{1}$ chooses a random $\theta \in Z_{q}$, sets $\sigma_{1}=\theta$ and computes
  $$C_{ji}^{*}=h_{ji}^{s_{1}}\cdot g^{\sigma_{1}w_{ji}^{*}}=g^{\eta_{ji}\left(c+\varphi \right)+\theta w_{ji}^{*}}.$$
(2) For $j=\hat{j}$, $B_{1}$ chooses a random $\theta \in Z_{q}$, sets $\sigma_{1}=bc+\theta$ and computes
  $C_{\hat{j}i}^{*}=h_{\hat{j}i}^{s_{1}}\cdot g^{\sigma_{1}w_{\hat{j}i}^{*}}=g^{-w_{\hat{j}i}^{*}b\varphi +\eta_{\hat{j}i}\left(c+\varphi \right)+\theta w_{\hat{j}i}^{*}}$.

- **Registration Queries II.**$A_{1}$ submits a polynomially bounded number of registration queries with respect to attribute sets ${\vec{z}}_{1},...,{\vec{z}}_{q}$. $B_{1}$ responds as it did in **Registration Queries I**.
- **Guess.**$A_{1}$ outputs a guess $b^{\prime }$ of *b*. If $b^{\prime }=b$, $B_{1}$ will guess *T* is a DDH tuple, i.e., $\bar{b}=0$; otherwise, it guesses *T* is a random tuple, i.e., $\bar{b}=1$. It indicates that if the adversary wins this game with nonnegligible advantage, then the simulator will have obviously advantage in the DDH game.

**Theorem** **2.** *Assume the CDH assumption holds, then the proposed zk-MIPE scheme is one-time authenticate.*

**Proof.**  Against an adversary who wants to forge a valid proof for the attributes he/she does not possess, the security algorithms are designed as follows.

In our scheme, for each task, the value of a tag $S=e{\left(g,g\right)}^{\frac{1}{\phi +H(C_{0})}}$ submitted by a user *u* is different and unique fixed. If submitting a tag twice will be forbidden. Thus, as follows, we show that it is difficult for unsuitable workers to forge a valid authentication message based on the CDH assumption. □

**Algorithm II**

- **Init.** The challenger sets $\vec{G}=\left(G_{1},G_{T}\right)$ and randomly chooses $\left(g,g^{a},g^{b}\right)\in G_{1}$. Then it sends $g,g^{a},g^{b}$ to $B_{2}$. After that, $A_{2}$ submits the challenge access structure and message $\left({\vec{w}}^{*},M^{*}\right)$.
- **CA Setup.** Running the CA setup algorithm, $B_{2}$ does as in Algorithm I.
- **AA Setup.** Running the AA setup algorithm, $B_{2}$ does as in Algorithm I.
- **Registration Queries I.** Running the registration algorithm, $B_{2}$ does as in Algorithm I.
- **Verification Queries I.**$A_{2}$ submits a series of queries about $\left(M_{k},{\vec{w}}_{k},{\vec{z}}_{k}\right)$ to $B_{2}$. It requires that ${\vec{w}}_{k}\neq {\vec{w}}^{*}$, $<{\vec{w}}_{k},{\vec{z}}_{k}>=0$ and $<{\vec{w}}^{*},{\vec{z}}_{k}>\neq 0$, and if not, it aborts. $B_{2}$ runs matching and detection verification algorithm, interacts with $A_{2}$, and generates proof transcript for $\left(M_{k},{\vec{w}}_{k},{\vec{z}}_{k}\right)$.
- **Forgery.** For the specified $\left(M^{*},{\vec{w}}^{*}\right)$, $A_{2}$ chooses a worker public key $U^{*}$ and an attribute vector ${\vec{z}}^{*}$ such that $<{\vec{w}}^{*},{\vec{z}}^{*}>=0$. In this algorithm, we will not consider the privacy of ${\vec{w}}^{*}$. Based on ${\vec{w}}^{*}$, $B_{2}$ computes ciphertext about message $M^{*}$. Then $A_{2}$ interacts with $B_{2}$ to generate a transaction of the protocol $P_{0}$, proving that it has the private key about a suitable vector. If $A_{2}$ outputs a valid forged proof and the protocol is sound, $B_{2}$ could then obtain $g^{ab}$ from the forgery.

**Theorem** **3.** *Suppose that the q-DDHI assumption holds and the protocol $P_{0}$ is zero-knowledge, then the proposed scheme is private and unlinkable.*

**Proof.**  To prove the privacy of the scheme, we first summarize the zero-knowledge of $P_{0}$.

*Zero-knowledgeness of $P_{0}$*. For the implementation of $P_{0}$, we introduced some auxiliary inputs $\left(\pi_{T},\chi_{T},\pi_{C},{\left\{\pi_{A_{j}},\pi_{Q_{ji}}\right\}}_{j\in [1,m],i\in [1,n]}\right)$ and protocol $P_{1}$. Based on the Logarithm assumption and the DDH assumption, the zero-knowledge property of $P_{1}$ is guaranteed for honest verifier, i.e., there exists a simulator S on imputing a random challenge ε, the simulator could output a transcript for $(L_{1},...,L_{5},z_{\phi }$, $z_{\kappa },z_{\delta }$,$z_{\mu },z_{v_{1}}$,...,$z_{v_{m}}$,$z_{\varsigma_{11}}$,...,$z_{\varsigma_{mn}}$,$z_{\rho_{1}}$,$z_{\rho_{2}})$. For any adversary, the distribution of the output is indistinguishable. By invoking S the simulator of protocol $P_{1}$, protocol $P_{0}$ could further prove its zero-knowledge property. □

Then we define the game between an adversary $A_{3}$ and a simulator $B_{3}$ which is given a *q*-DDHI instance as follows.

**Algorithm III**

- **Init.** The challenger sets $\vec{G}=\left(G_{1},G_{T}\right)$ and randomly chooses $g,g^{x},g^{x^{2}},...,g^{x^{q}}\in G_{1}$. It flips a coin $\bar{b}$. If $\bar{b}=0$, it computes $T=e{\left(g,g\right)}^{\frac{1}{x}}$; otherwise, it chooses a random $T\in G_{T}$. After that, $A_{3}$ submits two challenge users $u_{0},u_{1}$ with attribute vector $\vec{z_{0}}$, $\vec{z_{1}}$ to $B_{3}$.
- **CA Setup.** Given the secure parameter λ, $B_{3}$ chooses a random $t\in Z_{p}$ and sets $Y=g^{t}$. Then it gives the public key $PK=\{Y,H_{1}\}$ to $A_{3}$.
- **AA Setup.**$B_{3}$ randomly chooses ${\left\{x_{j},\eta_{ji}\right\}}_{j\in [1,m],i\in [1,n]}$, sets $\alpha_{j}=x_{j}$, $\gamma_{ji}=\eta_{ji}$ and lets $SK_{j}={\left\{\alpha_{j},\gamma_{ji}\right\}}_{i\in [1,n]}$ for $AA_{j}$. Then it computes $h_{ji}=g^{\eta_{ji}}$ and sends the public key $PK_{j}{=\{e\left(g,g\right)}^{\alpha_{j}},$$h_{j1},...,h_{jn}\}$ to $A_{3}$.
- **Registration Queries I.**$B_{3}$ sets $CK_{u^{*}}=g^{x}$ for a user $u^{*}$ and receives the value $T_{u^{*}}$, which may equal to $g^{\frac{1}{x+1}}$ or a random element in $G_{1}$, from the challenger initially. $A_{3}$ issues registration queries repeatedly. $B_{3}$ generates the secret key honestly except for $u^{*}$. If $u_{i}=u^{*}$, it aborts. Moreover, it is required that $A_{3}$ does not make secret key queries for both $u_{0}$ and $u_{1}$.
- **Challenge.** Without loss of generality, $B_{3}$ assumes $u_{0}=u^{*}$. It flips a coin b∈{0,1} and runs registration queries to obtain the corresponding $K_{u_{b}}$. Then, $B_{3}$ operates **Task Releasing** with an attribute vector $\vec{w^{*}}$ (with restrictions that $<\vec{w^{*}},\vec{z_{0}}>=0$ and $<\vec{w^{*}},\vec{z_{1}}>=0$) to acquire the ciphertext $C^{*}$. After receiving $C^{*}$, $A_{3}$ issues **Verification** and receives a valid proof from $B_{3}$ by applying the zero-knowledge protocol $P_{0}$.
- **Registration Queries II.**$A_{3}$ submits a polynomially bounded number of registration queries repeatedly. $B_{3}$ responds as it did in **Registration Queries I**.
- **Guess.**$A_{3}$ outputs a guess $b^{\prime }$ of *b*. If $b^{\prime }=b$, $B_{3}$ will guess *T* is a *q*-DDHI tuple, i.e., $\bar{b}=0$; otherwise, it guesses *T* is a random tuple, i.e., $\bar{b}=1$. Observe that if *H* is a one-way pseudo-random hash function and the *q*-DDHI assumption holds, the adversary will know nothing about $\beta_{u}$. By the zero-knowledge property of protocol $P_{0}$, the information about the identity *U*, the policy $\vec{w}$ and the attribute $\vec{z}$ will not be leaked. Thus, the algorithm could protect identity privacy and submission unlinkability.

## 7. Performance Evaluation

In reality, we implement the ZK-MIPE scheme on a Linux desktop with 6-core Intel(R) Xeon(R) Platinum 8369HC CPU 3.40 GHz processor and 32 GB of RAM. We use the PBC library to simulate the group operations. The symmetric elliptic curve SS512 is chosen with embedding degree 2 and a 512-bit base field.

Table 1 and Table 2 show the comparison between our scheme and other solutions in terms of functionality and security. Compared with [24], zk-MIPE supports more flexible matching poly and supports worker identity privacy. Compared with [23,25], zk-MIPE provides privacy for task constraints and worker attributes. As follows, we analyze the computational complexity of each participant in our scheme and test the running time to demonstrate scheme’s effectiveness. The notations applied in the proposed scheme are summarized in Table 3. Ignoring the operations of equality comparison, hash and multiplication, the communication and computation comparison of the schemes is shown in Table 4 and Table 5.

In our scheme, the main overhead on CA and $AA_{j}$ are from system setup and user registration. In CA setup, the computation complexity of CA is $E_{1}$. In AA setup, the computation complexity of $AA_{j}$ is $nE_{1}+P$. In user registration, the computation complexity of CA and $AA_{j}$ are $2kE_{1}$ and $3kE_{1}$, respectively. The total communication complexity of the authorities for distributing a key to a registered user is $m(Z_{q}+3|G_{1}|)$.

**Figure 3:**
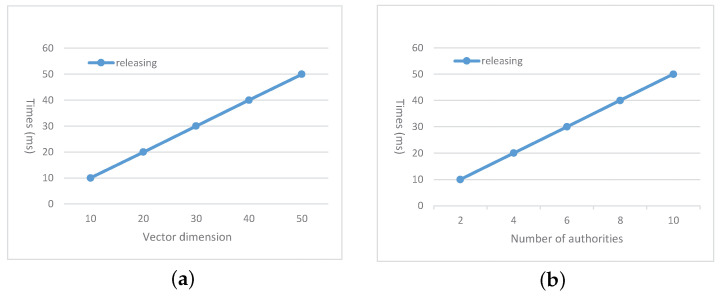
Time cost on the requester (**a**) under different values of *n* where m=5; (**b**) under different values of *m* where n=20.

The main overhead on the requester is from task releasing. In this step, the requester expresses the task requirements with vector $\vec{w}$ and encrypt the task based on $\vec{w}$ such that only the suitable worker could decrypt the task content. Meanwhile, the requester is required to blind the vector $\vec{w}$ for the CS to perform matching verification in the matching and submission verification phase. The computation complexity of the requester is $\left(4+4nm\right)E_{1}+2E_{T}$. The total communication complexity of the requester for task releasing is $\left(5+2nm\right)|G_{1}\left|+\right|G_{2}|$. To test the time cost of the requester, we set the number of attribute authorities as m=5, and vary the number of attributes *n* in Figure 3a. In Figure 3b, we set n=20 and vary the number of attribute authorities *m*.

**Figure 4:**
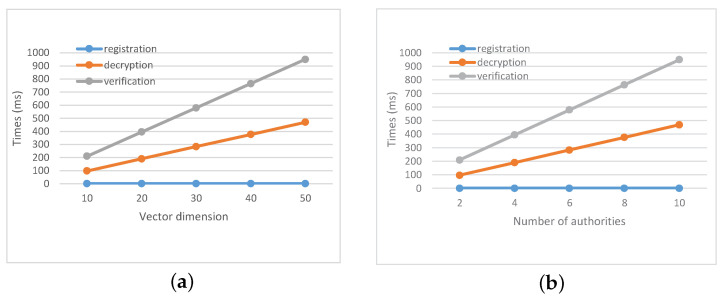
Time cost on the worker (**a**) under different values of *n* where m=5; (**b**) under different values of *m* where n=20.

The main overhead on the worker is from registration, decryption and verification. As shown in Figure 4a, we set m=5, and vary the number of attributes to test the time cost on the worker. In Figure 4b, we set n=20 and vary the number of attribute authorities. In user registration and decryption, the computation complexity of the worker is $E_{1}+nmE_{1}+\left(m+n+1\right)P$. Although in decryption algorithm, the computing cost for the worker increases linearly with the number of attributes, most of the computing overhead can be transferred to the CS by outsourcing computing. In this case, the worker only needs to carry out a small amount of calculation.

**Figure 5:**
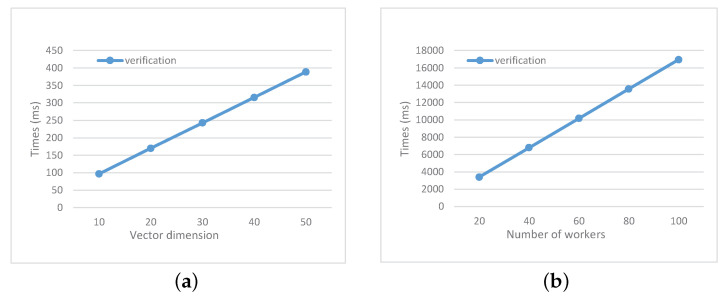
Time cost on CS (**a**) under different values of *n* where m=5 and k=1; (**b**) under different values of *k* where m=5 and n=20.

In the stage of submission and verification, the worker and CS achieve privacy-preserving matching and multi-submission verification through a zero-knowledge proof protocol. The interactive proof protocol consists of 3 rounds. The total computation and communication complexity of the worker are $\left(9+m+nm\right)E_{1}+\left(6+m+n\right)E_{T}+\left(4+n+m\right)P$ and $5|G_{T}\left|+\left(7+m+nm\right)(\right|z_{q}\left|+\right|G_{1}\left|\right)$ respectively. In Figure 5, we take m=5, and vary *n* as well as the number of workers *k* to test the time cost of verification for CS. The total computation and communication complexity of CS are $5E_{1}+\left(10+m+nm\right)E_{T}+\left(5+2m+2nm\right)P$ and $2|G_{1}\left|+\right|z_{q}|$, respectively.

## 8. Conclusions

In this paper, we present a novel multi-authorities inner-product encryption and one-time anonymous authentication scheme to realize privacy-preserving task-matching and multi-submission detection. In the system, both the user attributes and the number of submissions will be applied as authorization factors. By combining zero-knowledge proof technology and our anti-collusion multi-authorities inner-product encryption, the task confidentiality, worker attribute and unlinkability between different tasks participated by the same worker are guaranteed simultaneously. Moreover, the security of the scheme is proved based on bilinear difficulty assumptions and zero-knowledge of the protocol. For the sake of completeness, we finally analyze the function and efficiency of the scheme and show that it is practical for crowdsourcing environments. In addition to crowdsourcing privacy protection, our method could also play its role in the fields of searchable encryption, nearest neighbor search, fine-grained access control, electronic voting, electronic payment, and anonymous authentication.

In future work, we will continue to improve the algorithm itself and try to construct privacy protection schemes in a distributed crowdsourcing scenario without a trusted third party. Furthermore, we will study the integration of cryptography and other technologies, such as machine learning technology, to further improve the flexibility and efficiency of the solution.

## Figures and Tables

**Table 1:** Functional comparison.

Scheme	Authority	Matching Policy	Repetition Detection	Multi-Keyword
Fo-SDD	Single	Unlimited	×	×
SybSub	Single	Range	×	×
ZebraLancer	Distribute	Unlimited	×	×
zk-MIPE	Multiple	Inner-Product	×	×

**Table 2:** Security comparison.

Scheme	Task Content Privacy	Task Constraint Privacy	Identity Privacy	Attribute Privacy
Fo-SDD	*√*	×	×	×
SybSub	×	*√*	×	*√*
ZebraLancer	×	×	*√*	×
zk-MIPE	*√*	*√*	*√*	*√*

**Table 3:** Notations in Fo-SDD, SybSub, ZebraLancer and zk-MIPE.

Notations	Description
$E_{1},E_{T}$	Exponentiation on $G_{1}$ and $G_{T}$ respectively
$E_{2}$	Exponentiation in Paillier encryption
*P*	Pairing on $\left(G_{1},G_{1}\right)\to G_{T}$
${\tilde{C}}_{s},{\tilde{C}}_{a}$	Ciphertext based on AES and public key encryption, respectively
$C_{sc}$	Coding a task into a smart contract
${\tilde{E}}_{s},{\tilde{E}}_{a},{\tilde{E}}_{P}$	Symmetric encryption, asymmetric encryption and Paillier encryption
${\tilde{D}}_{s},{\tilde{D}}_{a},{\tilde{D}}_{P}$	Symmetric decryption, asymmetric decryption and Paillier decryption
$l_{1},l_{2},l_{3}$	Bit length of task, attribute and smart contract, respectively
*M*	NP machine used to prove membership of an instance *x* in a given NP language *L*
$t_{M},s_{M}$	Operations and computation space of *M* for the instance *x*
poly	Universal polynomial
λ	Security parameter
*N*	Product of two primes
*m*	Number of attribute authorities
*n*	Dimension of the attribute-based vector
*l*	Number of attributes managed by each authority
*k*	Number of suitable workers

**Table 4:** Communication cost.

Scheme	Requester/Publisher	CS/Contract	Worker/Subscriber	Fog Node
Fo-SDD	$|G_{1}\left|+\right|G_{2}\left|+\right|{\tilde{C}}_{s}|+l_{1}$	$k(|Z_{q}|+|G_{2}|+|{\tilde{C}}_{s}|+l_{1})$	$2|Z_{q}\left|+4\right|G_{1}\left|+2\right|{\tilde{C}}_{s}|$	$k(|Z_{q}|+4|G_{1}|+|{\tilde{C}}_{s}|+l_{1})$
SybSub	$l_{2}+\left|Z_{{}^{N^{2}}}\right|$	$l_{1}$	$2|G_{1}|+l_{2}+\left|Z_{N^{2}}\right|$	-
ZebraLancer	$l_{3}$	$k|{\tilde{C}}_{a}|$	$|{\tilde{C}}_{a}|+s_{M}\mathrm{poly}\left(\lambda \right)$	-
zk-MIPE	$\left(5+2nm\right)|G_{1}\left|+\right|G_{2}|$	$2k|G_{1}\left|+k\right|Z_{q}|$	$5|G_{T}\left|+\left(7+m+nm\right)(\right|Z_{q}\left|+\right|G_{1}\left|\right)$	-

**Table 5:** Computation cost.

Scheme	Requester/Publisher	CS/Contract	Worker	Fog Node
Fo-SDD	$2E_{1}+{\tilde{E}}_{s}$	$k(E_{1}+E_{2}+P+{\tilde{D}}_{s})$	$6E_{1}+2E_{T}+2P+{\tilde{D}}_{s}$	$E_{T}+{\tilde{D}}_{s}+k\left(3E_{1}+{\tilde{E}}_{s}\right)$
SybSub	$lE_{2}+2l{\tilde{E}}_{P}$	$kl\left(E_{1}+{\tilde{D}}_{P}\right)+l\left(k+1\right)P$	$3lE_{1}+2l{\tilde{E}}_{P}+lE_{2}$	-
ZebraLancer	$E_{1}+C_{sc}+t_{M}\mathrm{poly}\left(\lambda \right)$	kpoly(λ)	${\tilde{E}}_{a}+t_{M}\mathrm{poly}\left(\lambda \right)$	-
zk-MIPE	$\left(4+4nm\right)E_{1}+2E_{T}$	$5kE_{1}+\left(10+m+nm\right)kE_{T}+\left(5+2m+2nm\right)kP$	$\left(9+m+nm\right)E_{1}+\left(4+n+m\right)\left(E_{T}+P\right)+2E_{T}$	-
